# Association of Short-Term Increases in Ambient Fine Particulate Matter With Hospitalization for Asthma or COPD During Wildfire Season and Other Time Periods

**DOI:** 10.1016/j.chpulm.2024.100053

**Published:** 2024-03-29

**Authors:** Benjamin D. Horne, Mary M. Johnson, Denitza P. Blagev, Francois Haddad, Kirk U. Knowlton, Daniel Bride, Tami L. Bair, Elizabeth A. Joy, Kari C. Nadeau

**Affiliations:** Intermountain Medical Center Heart Institute (B. D. H., K. U. K., D. B., and T. L. B.), Salt Lake City, UT; the Division of Cardiovascular Medicine, Department of Medicine (B. D. H. and F. H.) and the Cardiovascular Institute (B. D. H. and F. H.), Stanford University, Stanford, CA; the Department of Environmental Health (M. M. J. and K. C. N.), Harvard T.H. Chan School of Public Health, Boston, MA; the Division of Pulmonary and Critical Care Medicine (D. B. P.), Department of Internal Medicine, Intermountain Health, Salt Lake City, UT; the Division of Cardiology (K. U. K.), Department of Internal Medicine, University of Utah, Salt Lake City, UT; the Wellness & Nutrition (E. A. J.), Intermountain Health, Salt Lake City, UT; and the Department of Family and Preventive Medicine (E. A. J.), University of Utah, Salt Lake City, UT.

**Keywords:** air pollution, air quality, temperature inversion, wildfire smoke

## Abstract

**BACKGROUND:**

Short-term increases in air pollution are associated with poor asthma and COPD outcomes. Short-term elevations in fine particulate matter (PM_2.5_) due to wildfire smoke are becoming more common.

**RESEARCH QUESTION:**

Are short-term increases in PM_2.5_ and ozone in wildfire season and in winter inversion season associated with a composite of emergency or inpatient hospitalization for asthma and COPD?

**STUDY DESIGN AND METHODS:**

Case-crossover analyses evaluated 63,976 and 18,514 patients hospitalized for primary discharge diagnoses of asthma and COPD, respectively, between January 1999 and March 2022. Patients resided on Utah’s Wasatch Front where PM_2.5_ and ozone were measured by Environmental Protection Agency-based monitors. ORs were calculated using Poisson regression adjusted for weather variables.

**RESULTS:**

Asthma risk increased on the same day that PM_2.5_ increased during wildfire season (OR, 1.057 per + 10 μg/m^3^; 95% CI, 1.019-1.097; *P* = .003) and winter inversions (OR, 1.023 per +10 μg/m^3^; 95% CI, 1.010-1.037; *P* = .0004). Risk decreased after 1 week, but during wildfire season risk rebounded at a 4-week lag (OR, 1.098 per +10 μg/m^3^; 95% CI, 1.033-1.167). Asthma risk for adults during wildfire season was highest in the first 3 days after PM_2.5_ increases, but for children, the highest risk was delayed by 3 to 4 weeks. PM_2.5_ exposure was weakly associated with COPD hospitalization. Ozone exposure was not associated with elevated risks.

**INTERPRETATION:**

In a large urban population, short-term increases in PM_2.5_ during wildfire season were associated with asthma hospitalization, and the effect sizes were greater than for PM_2.5_ during inversion season.

Ambient air pollution is well established as a risk factor for poor respiratory health outcomes, including for asthma and COPD.^1^ Air pollutants may affect health through short-term increases (ie, days to weeks) in ambient fine particulate matter (PM_2.5_), ozone, sulfur dioxide, and nitrogen dioxide, with individual and perhaps synergistic effects. Acute increases of pollutants are often caused by short-term environmental changes (eg, wildfires, summer heat waves, winter air stagnation). Exposure to short-term increases in PM_2.5_ is associated with incident asthma and hospitalizations for asthma,^2–12^ and with higher risk of COPD incidence and COPD exacerbation.^4,13–16^ Long-term exposure (ie, years) to air pollution, especially to PM_2.5_, is also associated with poor respiratory outcomes and mortality.^17–19^ In the American West, higher PM_2.5_ due to wildfires and higher ambient ozone has been found.^20,21^ Short-term PM_2.5_ increases due to local wildfire smoke were associated with health risks,^9,11,12,22^ and may be more deleterious than pollution from other sources.^23^

Air pollution exposures have been extensively studied on Utah’s Wasatch Front (eg, child respiratory health,^2^ COPD,^13^ acute respiratory infection^10^). The Wasatch Front acts as a natural laboratory for real-world evaluations of health outcomes because winter temperature inversions often trap air pollutants, exposing the population to dramatic short-term increases in PM_2.5_. The region’s baseline air quality shows low annual median PM_2.5_ of 6.5 μg/m^3^ and median ozone of 30 parts per billion (ppb). Acute short-term increases in pollutants permit the observation of population-wide health changes. Inversions do not occur in warm months. Wildfires, usually in coastal states, are the presumptive source of any short-term PM_2.5_ increases on the Wasatch Front during June through October against the baseline of other PM_2.5_ sources (eg, vehicles, industry). It is untested in the Wasatch Front population whether PM_2.5_ elevations during wildfire season (with possible concomitant ozone elevations) contribute to risks of major adverse respiratory events or what the magnitude of risks is during wildfire and wintertime inversion seasons.

This study evaluated the influences of short-term increases in ambient air pollution during wildfire season on acute changes in respiratory health in the Wasatch Front population due to wildfire smoke primarily from distant coastal fires and quantified the seasonal magnitude of risk. The primary aim was to examine the association of PM_2.5_ with hospitalizations for asthma and COPD, with the secondary aim of assessing ozone pollution associations.

## Study Design and Methods

### Population and Exposures

Patients had emergency or inpatient encounters for asthma or COPD at 11 Intermountain Health hospitals along Utah’s Wasatch Front from January 1, 1999, through March 31, 2022. For secondary outcomes of the first diagnosis of asthma or COPD, outpatient clinics also provided data. Intermountain provides health care for about 70% of the local population, and patients were unrestricted by age, sex, race, or other characteristics. Age, sex, race, and ethnicity were self-reported, and due to the large proportion of patients identifying as non-Hispanic White, those of other races and ethnicity were combined into one group. Data were gathered from the electronic health record. This study was approved by the Intermountain Institutional Review Board as a minimal-risk data-only examination of historical records with a waiver of consent.

About 80% of the 3.3 million Utahns reside on the Wasatch Front, an approximately 160-km (north to south) by 32-km (east to west) area of mountain valleys. Valley floors sit at 1,280 to 1,370 meters above sea level and mountain peaks ring the valleys, reaching 1,830 to 3,660 meters. The topography concentrates pollutants during winter temperature inversions in which pollutants from vehicular, point, and area sources are trapped in the valleys by high pressure aloft. Influxes of PM_2.5_ in summer and early fall from wildfire smoke most commonly arise from distant coastal wildfires. Furthermore, elevated ozone contributes to poor air quality in warm months.

Air pollution data were collected by monitors on the Wasatch Front, a region of concern historically due to winter air pollutant levels that exceeded US Environmental Protection Agency standards. Prior evaluations of regional health risks due to air pollution were performed primarily due to winter inversion-concentrated PM_2.5_ levels,^2,13^ but risk was evaluated across the entire year.^10^ This study focused on wildfire season because drought conditions from 2012 to 2022 elevated PM_2.5_ from wildfire smoke in summer and early fall. PM_2.5_ levels were measured daily since 1999, with daily ozone measurements since 2006 (only in May-October before 2006). Primary monitors (Hawthorne in Salt Lake City, Lindon in Provo/Orem, and Ogden [closed 2019]/Harrisville [opened 2019]) are located centrally with seven secondary monitors (Bountiful, Rose Park, Magna [closed 2017], Copperview [opened 2018], Herriman, North Provo [closed 2017], and Spanish Fork) across four counties where patients resided. Exposure estimates used the primary monitor closest to patient residences based on the centroid of zip codes. The median distance from residential zip codes to primary monitors was 14.42 km (25th and 75th percentiles, 7.23 and 19.89 km, respectively). Missing pollution data were imputed using the most highly correlated secondary site with available data (correlations with those sites were high, with *r*^2^ 0.73-0.93). For PM_2.5_, 1% of measurements were missing from all sites and 3% for ozone. Pollution data were obtained from the US Environmental Protection Agency Air Quality System Data Mart. Weather data were collected at the Salt Lake City International Airport by the US National Weather Service.

### Study Outcomes

The two primary outcomes of interest were hospitalization (ie, emergency visit, inpatient admission) for the primary diagnosis of asthma or hospitalization for the primary diagnosis of COPD. Analyses examined associations of PM_2.5_ with hospitalization separately during wildfire and inversion seasons. Secondary outcomes were first asthma diagnosis and first COPD diagnosis. Due to very low regional outmigration, the existence of electronic health records at Intermountain prior to 1999, and the broad catchment area, incident diagnoses were differentiated from repeated health care encounters (ie, prevalent disease). Diagnoses used International Classification of Diseases, Ninth Revision (ICD-9) or International Classification of Diseases, 10th Revision (ICD-10) codes for asthma (ICD-9: 493; ICD-10: J45) or COPD (ICD-9: 491, 492, 494, and 496; ICD-10: J41, J42, J43, J44, and J47). Local definitions of wildfire season (June 1-October 31) and inversion season (November 1-March 31) were used, with April and May used only in full-year analyses (this was not an evaluation of warm and cold seasons per se). Patients transferred between facilities were included based on just the date of admission to the first facility. Only the first hospitalization was included per patient due to influences of interventions and patient education on subsequent events.

### Statistical Methods

**Figure 1 –:**
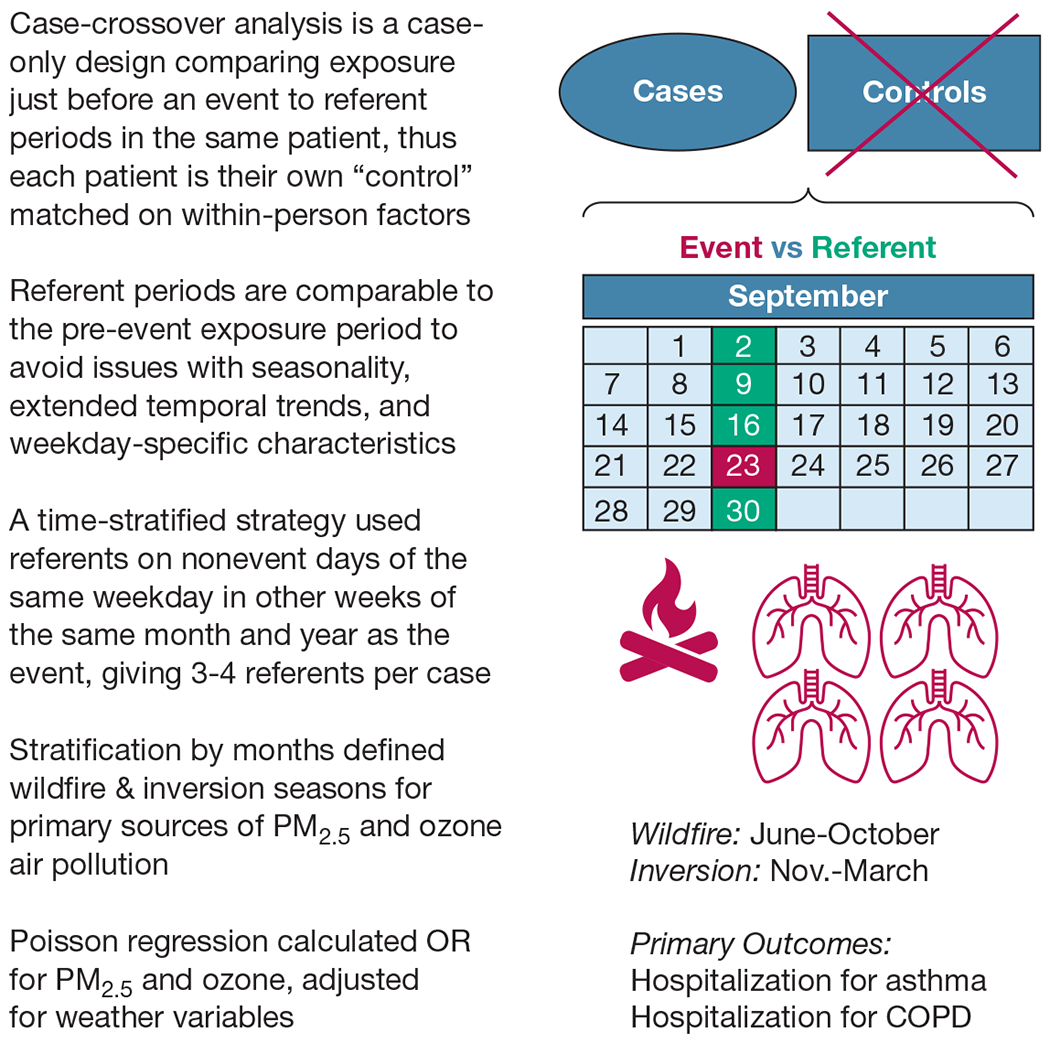
Graphical explanation of the case-crossover design. PM_2.5_ = fine particulate matter.

Analyses of air pollution associations with asthma and COPD outcomes used the time-stratified case-crossover method.^24,25^ Case-crossover is a case-only design that compares exposures occurring just before an event to referent (or control) periods when the event did not occur for that patient (Fig 1). Patients serve as their own control with all patient-specific factors matched by design. Referent days are chosen from the same day of the week within the same month and year to be comparable with the pre-event period because the exposure is shared as an ecological measure and exhibits seasonality, extended within-season temporal trends, and weekly trends (that are partly social and economic) based on the day of the week.^24,25^ Each event is matched to three or four control periods. Such confining to within-month analyses allowed separation of wildfire and inversion seasons.

Conditional Poisson regression using a quasi-Poisson distribution accounted for potential overdispersion.^26^ Base Poisson regression models used mean daily pollutant levels to evaluate asthma and COPD events on the concurrent day of pollution exposure (lag 0), for pollution level 1 day prior to the event (lag 1), lag 2, a moving average (mAvg) of mean pollution level for all lag days 0 to 2 (mAvg 0-2), concurrent day through 6 days prior (mAvg 0-6), the second prior week only (mAvg 7-13), the third prior week (mAvg 14-20), and the fourth prior week (mAvg 21-27). Squared terms for pollutants were also evaluated. Multivariable Poisson regression adjusted for minimum daily temperature, relative humidity, and barometric pressure. Other measures of temperature were modeled, but results for pollution were similar. ORs and 95% CIs were computed for associations with study outcomes of a +10-μg/m^3^ increment of PM_2.5_ or + 10-ppb increment of ozone. The gnm package in R statistical software (v 4.2.0, the R Foundation) was used for analyses.

Based on physiology and treatment plans, and priors from previous studies,^2,3,5–7^ it was expected that hospitalizations for asthma would be affected immediately by PM_2.5_ increases, whereas hospitalizations for COPD would require an exposure period of 1 week.^14^ The primary hypothesis for asthma risk was that concurrent day PM_2.5_ elevation is associated with hospitalization for the primary discharge diagnosis of asthma, with wildfire and inversion analyses tested at *P* ≤ .025 (Bonferroni-corrected for two seasons). The primary hypothesis for COPD risk was that PM_2.5_ mAvg 0 to 6 lag was associated with hospitalization for the primary discharge diagnosis of COPD (also tested at *P* ≤ .025). Subanalyses by age and sex, full-year analyses, and evaluations of first diagnosis of asthma or COPD were evaluated at *P* ≤ .05 as confirmatory or as exploratory hypotheses requiring future validation.

## Results

**Figure 2 –:**
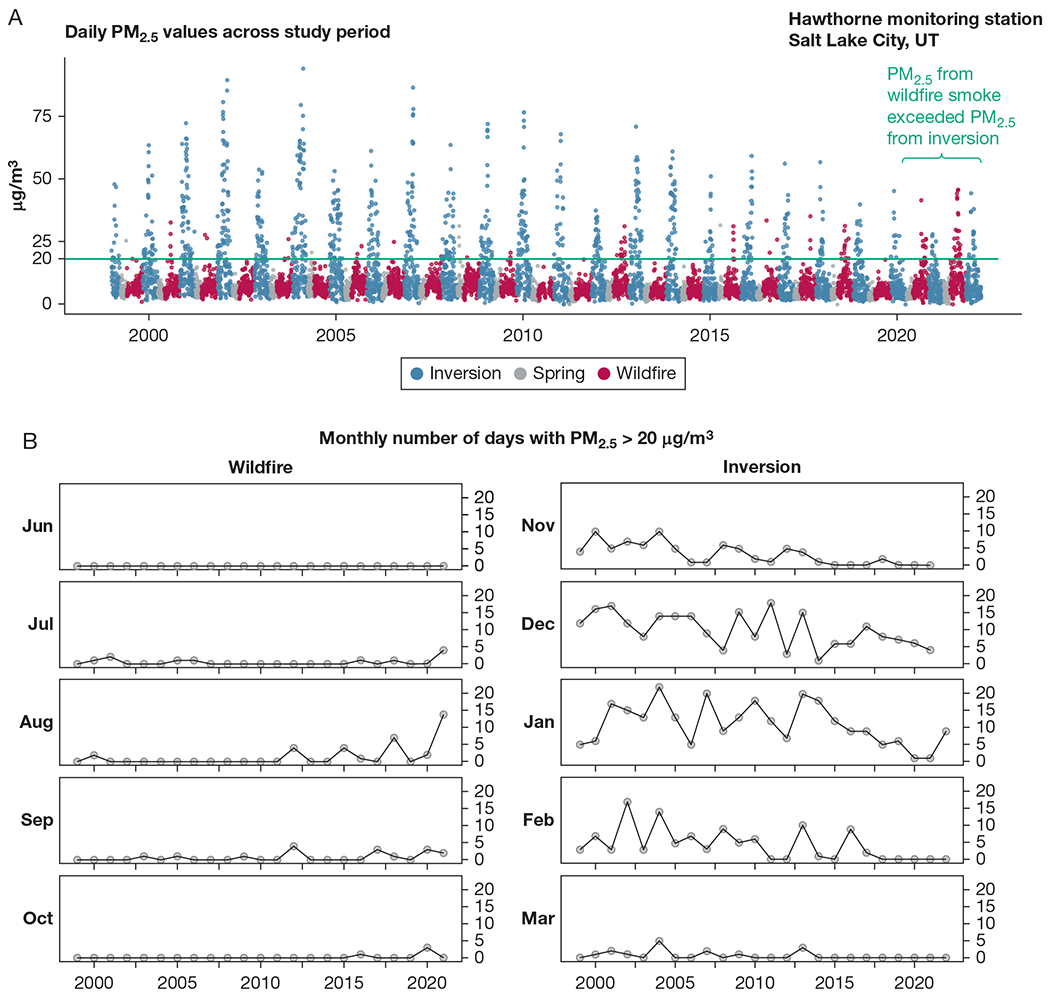
A, B, Air pollution data during the whole study period (January 1999-March 2022). A, PM_2.5_ showed that the levels during the inversion season (blue data points from November-March) dropped considerably over the period 2012 to 2022, whereas PM_2.5_ from wildfire smoke increased during wildfire season (red data points from June-October) over the same period (green line: threshold for elevated PM_2.5_ used in B). Gray data points are measured pollution levels during April and May. Evaluation of thresholds of air pollution showed in more dramatic fashion the trends over the years to have more days with (B) short-term increases of PM_2.5_ in wildfire season (and declines in PM_2.5_ during inversion season). PM_2.5_ = fine particulate matter.

PM_2.5_ (median, 6.5 μg/m^3^) and ozone (median, 30 ppb) over 23.25 years are shown in Figure 2A and e-Figure 1A, respectively, with median PM_2.5_ of 6.5 μg/m^3^ in wildfire season and 8.2 μg/m^3^ in inversion season. Median PM_2.5_ in 1999 through 2011 and 2012 through 2022 were, respectively, 6.8 and 6.1 μg/m^3^ in wildfire season and 10.5 and 5.9 μg/m^3^ in inversion season. Ozone was lower in winter and higher in summer. Figure 2B highlights declines in days with elevated PM_2.5_ during inversion season for 2012 through 2022 compared with 1999 through 2011. In contrast, more PM_2.5_ elevations (Fig 2B) and higher ozone (> 50 ppb) (e-Fig 1B) occurred in wildfire season in 2012 through 2022 compared with historically. During 2020 to 2022, PM_2.5_ elevations in wildfire season exceeded that of inversion season (Fig 2A).

Hospitalization for asthma was found for 63,976 patients over the 1999 to 2022 study period. Patients’ average age was 22.6 years, 51.0% were female, 16.0% had hypertension, and 10.1% had a smoking history. Hospitalization for COPD was recorded for 18,514 patients, with patients’ age averaging 63.5 years, and 50.3% were female, 69.1% had hypertension, and 42.3% had a history of smoking. Other characteristics are included in Table 1. Secondary events of first asthma diagnosis included 213,443 patients and of new COPD diagnoses included 87,713 patients (Table 1).

**Figure 3 –:**
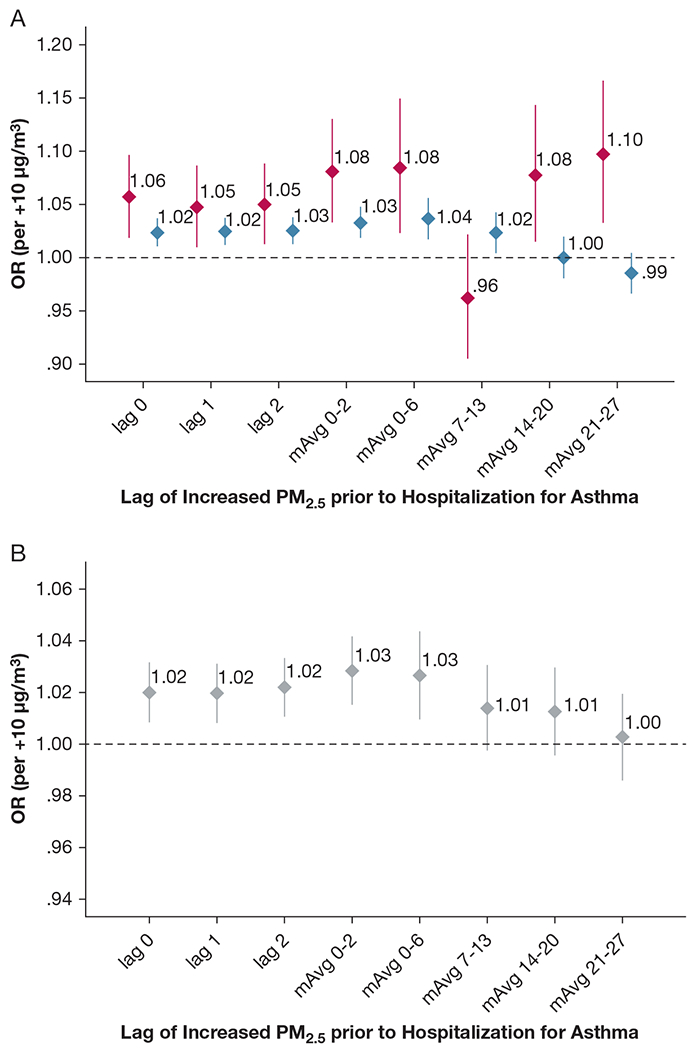

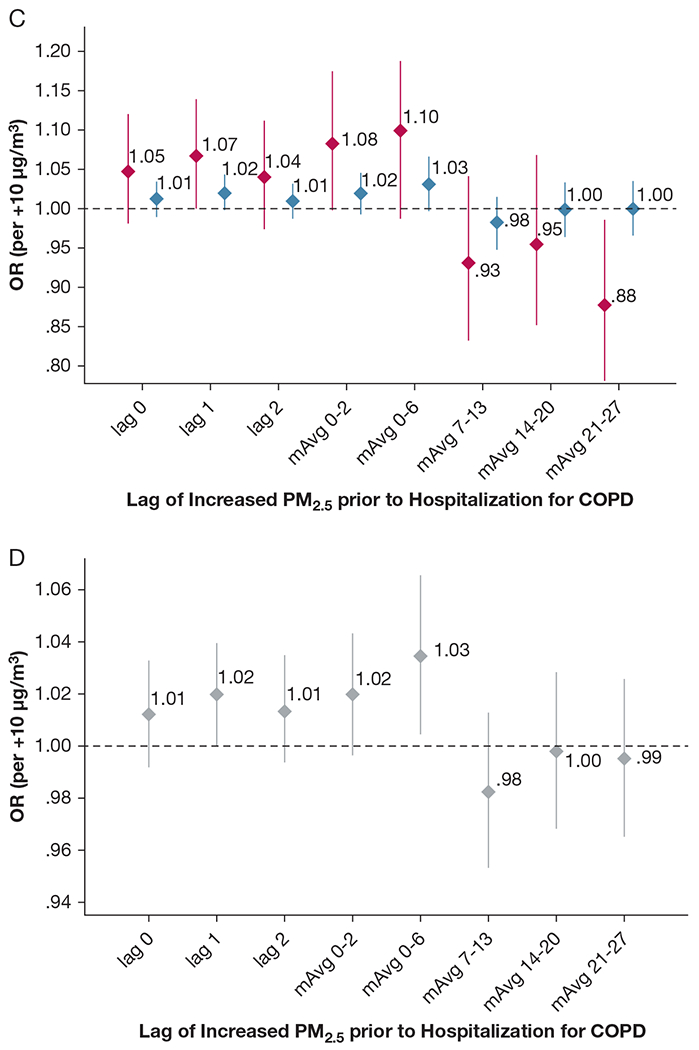
A-D, Forest plots showing the association of short-term increases (over days) in PM_2.5_ with (A) hospitalization for asthma during wildfire season (red) and inversion season (blue), (B) hospitalization for asthma across all months of the calendar year, (C) hospitalization for COPD during wildfire season (red) and inversion season (blue), and (D) hospitalization for COPD during the full year. Data are ORs (diamonds) and 95% CIs (whiskers). The x-axis provides the various days of lag examined between exposure and events. mAvg = moving average; PM_2.5_ = fine particulate matter.

Short-term increases in PM_2.5_ were associated with increased risk of hospitalization for asthma (Fig 3A) for lag 0 during wildfire season (OR, 1.057 per +10 μg/m^3^; 95% CI, 1.019-1.097; *P* = .003) and inversion season (OR, 1.023 per +10 μg/m^3^; 95% CI, 1.010-1.037; *P* = .0004). For wildfire season, PM_2.5_ lag 1 and lag 2 were associated with asthma hospitalization (*P* = .013 and *P* = 0008, respectively), whereas effect sizes were about one-half for lag 1 (*P* = .0002) or lag 2 (*P* = .0001) for inversion season. PM_2.5_ association with asthma hospitalization was significant for mAvg 0 to 2 and mAvg 0 to 6 in wildfire and inversion seasons (Table 2), with effect sizes more than double in wildfire vs inversion season. The pattern of association diverged at week 2 with elevated risk only for mAvg 7 to 13 lag in inversion season. For wildfire season, mAvg 14 to 20 and mAvg 21 to 27 were associated with risk elevations. Addition of squared terms increased ORs of linear terms (eg, linear terms in wildfire season had *P* < .05 and OR, 1.09, 1.09, 1.15, and 1.23 for lag 0, lag 2, mAvg 0-2, and mAvg 0-6, respectively), but the squared terms were not significant. Overall analysis of all months (Fig 3B) found a concurrent day rise in PM_2.5_ was associated with asthma hospitalization (OR, 1.020 per +10 μg/m^3^; 95% CI, 1.009-1.032; *P* = .0006), and risk elevations occurred across the first week (Table 2).

Short-term increases in PM_2.5_ had weak trends for COPD hospitalization for lag 1, mAvg 0 to 2, and mAvg 0 to 6 during wildfire season, and for lag 1 and mAvg 0 to 6 in inversion season (Fig 3C), but had *P* > .05 to .08. Seasonal results tracked with the overall mAvg 0 to 6 (Fig 3D) (OR, 1.035 per +10 μg/m^3^; 95% CI, 1.004-1.066; *P* = .025) and weak trends for lag 1 (*P* = .06) and mAvg 0 to 2 (*P* = .10). No other overall or season-specific PM_2.5_ association with COPD hospitalization was found (Table 2). Squared terms in COPD models were not significant and did not improve linear term ORs. Short-term increases in ozone were not associated with asthma or COPD hospitalization (e-Table 1). e-Appendix 1 provides further results by age and sex strata for ozone and for first diagnosis of asthma or COPD.

## Discussion

### Summary

This study found elevated asthma hospitalization risk during wildfire and inversion seasons in association with short-term increases in PM_2.5_, with effect sizes during wildfire season being higher than in inversion season. Risk increased on the day of PM_2.5_ elevation and was present for all lags in the first week in both seasons. Risk patterns diverged, however, after a 2-week lag, with risk tapering off during inversion season, whereas risk in wildfire season was elevated for 3- and 4-week lags. Risk patterns differed for adults and children and between female and male participants (e-Appendix 1). Weak association of short-term increases in PM_2.5_ with COPD hospitalization was found for 1-week lags across all months. Ozone increases were not associated with elevated risks of asthma or COPD (e-Appendix 1).

### Air Pollution From Wildfires

An expanding number of studies are addressing asthma or COPD in regions or time frames with short-term PM_2.5_ exposure due to wildfires. Changes in the climate necessitate a better understanding of the impacts of PM_2.5_ from wildfire and the effect of potentially unexpected and varying patterns of risk.^21^ This was shown in dramatic fashion in 2023 when unprecedented wildfires in Canada produced substantial PM_2.5_ exposures many hundreds of kilometers away in the United States. Those wildfires resulted in acute increases in emergency admissions for asthma, peaking at the highest risk on the second day of exposure in New York City.^27^ Such evaluations can improve understanding of the critical health risks from exposures to wildfire smoke at substantial distances and inform about time frames for onset and worsening of health.^28^ In part, such understanding can guide research evaluating inflammasome and other biomarkers of physiologic responses to inhaled pollutants and decision-making by individuals and physicians.

Short-term elevations in PM_2.5_ from wildfires in San Francisco, California, were previously linked to respiratory health risks.^22^ Asthma was affected by wildfire smoke, with relative risks more elevated than for COPD.^22^ In a study from Fresno, California, epigenetic changes were noted in children with and without asthma.^9^ Separately, children from that region who were exposed to PM_2.5_ from a 2015 wildfire had greater methylation of *Foxp3* and lower Th1 pro-inflammatory T cell levels compared with minors exposed to PM_2.5_ from a prescribed burn.^11^ Another study found that IL-1β and C-reactive protein were increased by wildfire exposure.^12^ In a recent study of wildfires in Washington state, the risk of emergency admissions locally for asthma and general respiratory diagnoses were found on the concurrent day of elevated PM_2.5_ and in the subsequent 5 days.^29^ Collectively, these findings reveal adverse health effects of pollutants generated by wildfires and address the consequences of wildfires that are, for example, more widespread and intense than prescribed fires. Importantly, these studies were close geographically to the source of wildfire smoke.^9,11,12,22,29^

### Behavioral Responses to Wildfire Smoke

This study reveals the need to better understand the risk of respiratory outcomes due to wildfire smoke exposures originating from distant sources.^27^ In particular, the risk of poor asthma outcomes here was profoundly higher in association with PM_2.5_ during wildfire season. Although these risks are expected to arise in part from physiologic responses to air pollutants, behavioral choices are also involved in the process of seeking care. Similar to other studies using clinical records, a decision to call for emergency transport or to otherwise travel to a health care facility is required for hospitalization to occur. Those decisions involve self-assessment of health needs. More research is needed regarding decisions and interventions that increase or decrease exposures or health outcomes during short-term elevations in air pollution, which this study did not evaluate. However, in addition to the typical increase in asthma medication use in the winter to proactively manage viral-induced asthma, we suggest that anticipatory guidance be given to patients during wildfire season to mitigate asthma risk by being prepared for symptoms, carrying appropriate medications, and anticipating staying indoors when outdoor smoke levels are high.

### Limitations

The observational design of this study limits causal interpretation because of the potential for residual uncontrolled confounding. The case-crossover design, however, controlled most individual characteristics and behaviors. This may not include one-time choices during an event or referent period. A wildfire source of all acute summer and early fall short-term elevations in PM_2.5_ was not documented, but is a reasonable assumption given the known regional sources for acute increases. Also, measurement of air pollutants was based on location of residence and not individual exposure, thus some misspecification may have occurred. With some longer lag structures, a potential overlap of inversion season analyses for November with pollution levels in October exists, but air pollution levels were low in October, especially compared with other months of the inversion season. Although many inversions last just a few days, a more prescient seasonal difference is that for an extended inversion, it takes multiple days to a week for PM_2.5_ levels to plateau, whereas pollution from wildfires usually lasts only a few days, thus seasonal exposure patterns may differ. Additionally, the background level of ozone in each month had only minimal daily changes and may have limited the ability to detect risk differences for large acute changes. The low racial and ethnic diversity in this population may limit generalization of these results to other regions because rates of asthma may be lower in this region, thus wildfire smoke and other sources of air pollutants may have greater effect in other populations, which requires further investigation. Another limitation is that socioeconomic measures were not available for use in these analyses. It was unknown what level of knowledge people in the community had of air quality events and methods of avoidance of wildfire exposure, or the prevalence of employment resulting in occupational exposures, thus measures of risk here may underestimate the effect of PM_2.5_ on respiratory outcomes. Finally, for the secondary outcomes of first asthma or COPD diagnosis, it is possible that some people had their initial diagnosis prior to 1999 or moved to the area after being diagnosed, but these should constitute a minority of cases. Overall, although background levels of air pollution are from standard human sources, short-term increases in PM_2.5_ during the summer and early autumn were primarily from wildfire smoke, and validation of the source of PM_2.5_ during that time frame being solely from wildfire smoke was not performed.

## Interpretation

Short-term increases in PM_2.5_ during both wildfire and inversion seasons were associated with hospitalization for asthma, but only marginally associated with COPD hospitalization. The strength of association for asthma was greater in wildfire season, with notably higher effect sizes that may be due to both physiologic and behavioral factors (which cannot be separated here). Similar results were found for the association between increases in PM_2.5_ and the first diagnosis of asthma or COPD. Particulate matter from wildfires is an increasing global problem, and this study suggests that it poses health risks to people living at a distance who are exposed to wildfire smoke.

## Supplementary Material

1

## Figures and Tables

**TABLE 1]:** Baseline Characteristics of Populations Studied for Asthma and COPD Outcomes

Asthma Events	Wildfire Season (June-October)	Inversion Season (November-March)	April and May
Emergency or inpatient admission for the primary diagnosis of asthma			
Asthma hospitalizations	23,918	29,027	11,031
Age, y	22.1 ± 20.9	23.2 ± 22.2	22.2 ± 21.6
Sex, male	11,962 (50.0)	13,916 (47.9)	5,441 (49.3)
Race, non-Hispanic White^a^	18,970 (79.3)	23,290 (80.2)	8,799 (79.8)
Hypertension history	3,572 (14.9)	4,957 (17.1)	1,730 (15.7)
Smoking history	2,554 (10.7)	2,878 (9.9)	1,020 (9.2)
First diagnosis of asthma			
Asthma diagnoses	79,850	98,308	35,285
Age, y	31.4 ± 23.3	30.8 ± 24	31.1 ± 24
Sex, male	35,080 (43.9)	43,676 (44.4)	15,647 (44.3)
Race, non-Hispanic White^a^	68,703 (86.0)	84,942 (86.4)	30,369 (86.1)
Hypertension history	16,161 (20.2)	19,717 (20.1)	7,280 (20.6)
Smoking history	5,956 (7.5)	7,312 (7.4)	2,435 (6.9)
COPD Events	Wildfire Season (June-October)	Inversion Season (November-March)	April and May
Emergency or inpatient admission for the primary diagnosis of COPD			
COPD hospitalizations	6,710	8,755	3,049
Age, y	63.3 ± 13.6	63.6 ± 13.7	63.4 ± 14
Sex, male	3,272 (48.8)	4,393 (50.2)	1,536 (50.4)
Race, non-Hispanic White^a^	5,958 (88.8)	7,823 (89.4)	2,699 (88.5)
Hypertension history	4,654 (69.4)	6,060 (69.2)	2,074 (68.0)
Smoking history	2,826 (42.1)	3,749 (42.8)	1,264 (41.5)
First diagnosis of COPD			
COPD diagnoses	34,386	38,533	14,794
Age, y	62.9 ± 16.5	62.4 ± 17.1	62.6 ± 17.1
Sex, male	17,991 (52.3)	20,211 (52.5)	7,765 (52.5)
Race, non-Hispanic White^a^	30,522 (88.8)	34,102 (88.5)	13,078 (88.4)
Hypertension history	20,499 (59.6)	22,617 (58.7)	8,800 (59.5)
Smoking history	8,904 (25.9)	9,874 (25.6)	3,664 (24.8)

Values are mean ± SD, No. (%), or No.

a Patients of other races were combined due to the relatively small proportion of each in the population. These included self-reported race and ethnicity by participants identifying as Hispanic White, American Indian or Alaska Native, Asian, Black or African American, or Native Hawaiian or Other Pacific Islander.

**TABLE 2]:** ORs (With 95% CIs) for the Association of Ambient PM_2.5_ Air Pollution With Hospitalization in an Emergency or Inpatient Setting for the Primary Diagnosis of Asthma or COPD

Pollutant/Event	Lag	Wildfire Season	Inversion Season	All Months
		(June-October)	(November-March)	(January-December)
PM_2.5_ air pollution (ORs and 95% CI are per +10 μg/m^3^)				
Asthma hospitalization (emergency or inpatient)				
	Lag 0	**1.057** (1.019-1.097)^a^	**1.023** (1.010-1.037)^b^	**1.020** (1.009-1.032)^b^
	Lag 1	**1.048** (1.010-1.087)^c^	**1.024** (1.011-1.037)^b^	**1.020** (1.008-1.031)^b^
	Lag 2	**1.050** (1.013-1.089)^c^	**1.025** (1.013-1.038)^b^	**1.022** (1.011-1.034)^b^
	mAvg 0-2	**1.081** (1.033-1.130)^b^	**1.033** (1.018-1.048)^b^	**1.029** (1.015-1.042)^b^
	mAvg 0-6	**1.084** (1.023-1.150)^b^	**1.036** (1.017-1.056)^b^	**1.027** (1.010-1.044)^b^
	mAvg 7-13	0.962 (0.905-1.022)	**1.023** (1.004-1.043)^c^	1.014 (0.998-1.031)
	mAvg 14-20	**1.078** (1.015-1.143)^c^	1.000 (0.981-1.020)	1.013 (0.996-1.030)
	mAvg 21-27	**1.098** (1.033-1.167)^a^	0.985 (0.966-1.005)	1.003 (0.986-1.020)
COPD hospitalization (emergency or inpatient)				
	Lag 0	1.048 (0.980-1.120)	1.012 (0.989-1.036)	1.012 (0.992-1.033)
	Lag 1	1.068 (0.9996-1.140)	1.021 (0.998-1.044)	1.019 (0.999-1.040)
	Lag 2	1.041 (0.973-1.112)	1.010 (0.987-1.033)	1.014 (0.993-1.034)
	mAvg 0-2	1.083 (0.997-1.175)	1.019 (0.993-1.046)	1.020 (0.996-1.044)
	mAvg 0-6	1.100 (0.987-1.225)	1.031 (0.997-1.067)	**1.035** (1.004-1.066)^c^
	mAvg 7-13	0.932 (0.832-1.043)	0.982 (0.948-1.016)	0.982 (0.953-1.012)
	mAvg 14-20	0.955 (0.852-1.069)	0.998 (0.963-1.034)	0.998 (0.968-1.029)
	mAvg 21-27	**0.878** (0.780-0.987)	1.000 (0.965-1.036)	0.995 (0.964-1.026)

All ORs and 95% CI data are per +10 μg/m^3^ for PM_2.5_. All ORs where *P* ≤ .05 are bolded; results that are bolded had *P* ≤ .05 and *P* > .025 and were not considered significant after correction for multiple comparisons. PM_2.5_ = fine particulate matter; mAvg 0-2, mean pollution level for all lag days 0 to 2; mAvg 0-6, mean pollution level for all lag days 0 to 6; mAvg 7-13, mean pollution level for all lag days 7 to 13; mAvg 14-20, mean pollution level for all lag days 14 to 20; mAvg 21-27, mean pollution level for all lag days 21 to 27.

a *P* < .005.

b *P* < .001.

c *P* ≤ .025.

## Take-home Points

### Study Question:

Are short-term increases in fine particulate matter (PM_2.5_) and ozone in wildfire season and in winter inversion season associated with a composite of emergency or inpatient hospitalization for asthma and COPD?

### Results:

Asthma risk was increased in association with PM_2.5_ in both wildfire season and winter inversion season, but for COPD the PM_2.5_ association was weak. Neither outcome was associated with short-term changes in ozone.

### Interpretation:

Short-term increases in ambient PM_2.5_ during wildfire season from June through October and in inversion season from November through March were associated with asthma hospitalization.

## ABBREVIATIONS:

ICD-9: International Classification of Diseases, Ninth Revision
ICD-10: International Classification of Diseases, 10th Revision
mAvg: moving average
PM_2.5_: fine particulate matter
ppb: parts per billion
mAvg 0-2: mean pollution level for all lag days 0 to 2
mAvg 0-6: mean pollution level for all lag days 0 to 6
mAvg 7-13: mean pollution level for all lag days 7 to 13
mAvg 14-20: mean pollution level for all lag days 14 to 20
mAvg 21-27: mean pollution level for all lag days 21 to 27
