# Supplementary material for: Association of Short-Term Increases in Ambient Fine Particulate Matter With Hospitalization for Asthma or COPD During Wildfire Season and Other Time Periods
Source: CHEST Pulm. 2024 Mar 29;2(2):100053. doi: 10.1016/j.chpulm.2024.100053 (PMC11238943; doi:10.1016/j.chpulm.2024.100053)

**Supplemental Materials for the study:**

**Association of Short-Term Increases in Ambient Fine Particulate Matter with Hospitalization for Asthma or COPD during Wildfire Season and Other Time Periods**

Benjamin D. Horne, PhD, MStat, MPH^1,2,3^; Mary M. Johnson, MD, PhD^4^; Denitza P. Blagev, MD^5^; Francois Haddad, MD^2,3^; Kirk U. Knowlton, MD^1,6^; Daniel Bride, MS^1^; Tami L. Bair, BS^1^; Elizabeth A. Joy, MD, MPH^7,8^; Kari C. Nadeau, MD, PhD^4^

^1^Intermountain Medical Center Heart Institute, Salt Lake City, UT; ^2^Division of Cardiovascular Medicine, Department of Medicine, Stanford University, Stanford, CA; ^3^Cardiovascular Institute, Stanford University, Stanford, CA; ^4^Department of Environmental Health, Harvard T.H. Chan School of Public Health, Boston, MA; ^5^Division of Pulmonary and Critical Care Medicine, Department of Internal Medicine, Intermountain Health, Salt Lake City, UT; ^6^Division of Cardiology, Department of Internal Medicine, University of Utah, Salt Lake City, UT; ^7^Wellness & Nutrition, Intermountain Health, Salt Lake City, UT; ^8^Department of Family and Preventive Medicine, University of Utah, Salt Lake City, UT.

**Supplemental Results**

In subanalyses stratified by age ≥18 years vs. <18 years, adults (Supplemental Figure S2A) had early elevation of risk of asthma hospitalizations in wildfire and inversion seasons (all ORs are per +10 μg/m^3^) at lag 0 (wildfire: OR=1.082, p=0.002; inversion: OR=1.025, p=0.008), lag 1 (wildfire: OR=1.055, p=0.036; inversion: OR=1.025, p=0.007), lag 2 (wildfire: OR=1.054, p=0.037; inversion: OR=1.031, p<0.001), mAvg 0-2 (wildfire: OR=1.102, p=0.002; inversion: OR=1.037, p<0.001), and mAvg 0-6 (wildfire: OR=1.078, p=0.07 [trend]; inversion: OR=1.042, p=0.003). In contrast, in children (Supplemental Figure S2B) the risk of asthma hospitalization was only elevated on the concurrent day (lag 0) (OR=1.022, p=0.013), lag 1 (OR=1.023, p=0.008), lag 2 (OR=1.020, p=0.019), and mAvg 0-2 (OR=1.029, p=0.004) in inversion season. It continued in inversion season with mAvg 0-6 (OR=1.031, p=0.016) and mAvg 7-13 (OR=1.034, p=0.009), but for children in wildfire season the asthma hospitalization risk was elevated only for the 1^st^, 3^rd^, and 4^th^ weeks after pollution elevation: mAvg 0-6 (OR=1.091, p=0.039), mAvg 14-20 (OR=1.117, p=0.009), and mAvg 21-27 (OR=1.167, p=0.0003). For adults, the CI for mAvg 21-27 for asthma hospitalization did not overlap the odds ratio for children in wildfire season (CI: 0.946, 1.120 for adults vs. OR: 1.167 for children), and the CI for children did not overlap the OR for adults (CI: 1.073, 1.267 for children vs. OR: 1.029 for adults), indicating nominally statistically significantly different results between adults and children (with p≤0.05) and suggesting a possible interaction between air pollution exposure and children in asthma outcomes.(S1)

Sex-specific analyses provided intriguing differences, with males tending to experience higher risk of both asthma and COPD hospitalizations associated with short-term increases in PM_2.5_. The greater risk in males was especially noted in the wildfire season for both asthma and COPD. For asthma hospitalization, short-term increases in PM_2.5_ in wildfire season was only early for females (Supplemental Figure S3A), with associations (all ORs are per +10 μg/m^3^) at lag 0 (OR=1.056, p=0.033) and mAvg 0-2 (OR=1.067, p=0.036), while for males (Supplemental Figure S3B) the wildfire season risks stretched across the first week (lag 0: OR=1.059, p=0.035; lag 1: OR=1.064, p=0.021; lag 2: OR=1.058, p=0.033; mAvg 0-2: OR=1.096, p=0.005; mAvg 0-6: OR=1.099, p=0.027) and resurfaced more strongly at week 4 (mAvg 21-27: OR=1.156, p=0.0009). Winter inversion asthma hospitalization risks were similar between females (lag 0: OR=1.024, p=0.009; lag 1: OR=1.029, p=0.001; lag 2: OR=1.034, p=0.0001; mAvg 0-2: OR=1.038, p=0.0002; mAvg 0-6: OR=1.033, p=0.014) and males (lag 0: OR=1.023, p=0.014; lag 1: OR=1.019, p=0.042; mAvg 0-2: OR=1.027, p=0.013; mAvg 0-6: OR=1.040, p=0.004; mAvg 7-13: OR=1.038, p=0.007). For males, the CI for mAvg 21-27 for asthma did not overlap the odds ratio for females in wildfire season (CI: 1.061, 1.259 for males vs. OR: 1.042 for females), and the CI for females did not overlap the OR for males (CI: 0.957, 1.133 for females vs. OR: 1.156 for males), indicating nominally statistically significantly different results between males and females (based on p≤0.05) and suggesting a possible interaction between air pollution exposure and males in asthma care.(S1)

For COPD hospitalization, no wildfire or inversion season associations were found for females (Supplemental Figure S3C), while the overall association for PM_2.5_ with COPD hospitalization at mAvg 0-6 shown in Figure 3D (see main paper) was largely due to inversion season risk in males (mAvg 0-6: OR=1.051 per +10 μg/m^3^, p=0.028) and a trend in wildfire season results (mAvg 0-6: OR=1.141 per +10 μg/m^3^, p=0.06). Unexpectedly, associations of PM_2.5_ with COPD hospitalization in wildfire season were found among males (Supplemental Figure S3D) for lag 0 (OR=1.107 per +10 μg/m^3^, CI: 1.015, 1.206, p=0.020), lag 1 (OR=1.102 per +10 μg/m^3^, CI: 1.012, 1.199, p=0.025), and mAvg 0-2 (OR=1.134 per +10 μg/m^3^, CI: 1.020, 1.259, p=0.019). Further, these results for males at lag 0 were significantly different than the findings for females, with the female lag 0 CI (0.926, 1.099) not overlapping the OR (1.107) for males and the female lag 0 OR (1.010) not overlapped by the lag 0 CI computed for males (1.015, 1.206), with nominal p≤0.05 suggesting an interaction between air pollution exposure and males.(S1) These findings should be taken with caution due to the multiple subanalyses and lack of associations overall at those lags for COPD hospitalization but that may indicate an important finding among males with wildfire smoke exposure.

No significant association of ozone with asthma hospitalization (Supplemental Figure S4A) was found (see also Supplemental Table S1). Ozone had a marginal association with COPD hospitalization (Supplemental Figure S4B) for mAvg 7-13 in inversion season (OR=1.099 per +10 ppb, CI: 1.010, 1.195; p=0.029), while full-year analysis showed mAvg 7-13 had OR=1.061 (p=0.043) (Table 2). No ozone association with COPD hospitalization was found for wildfire season. Joint analyses entering PM_2.5_ and ozone in the same model confirmed no associations of ozone, but associations of increased PM_2.5_ with outcomes remained significant.

In analyses of the first diagnosis of asthma (Supplemental Figure S5A), risk for PM_2.5_ lags of 1 day, mAvg 0-2, mAvg 0-6, and mAvg 7-13 was higher in wildfire season with, respectively, (all OR are per +10 μg/m^3^) OR=1.029 (CI: 1.007, 1.050, p=0.008), OR=1.032 (CI: 1.006, 1.059, p=0.016), OR=1.043 (CI: 1.009, 1.079, p=0.012), and OR=1.047 (CI: 1.013, 1.083, p=0.007). In inversion season, no association of PM_2.5_ was found with first asthma diagnosis. Age-stratified analyses of first asthma diagnosis reflected earliest impact in adults during inversion season (at lag 0: OR=1.012, p=0.017), with diagnosis in adults during wildfire season at mAvg 0-2 (OR=1.036, p=0.024), mAvg 0-6 (OR=1.053, p=0.010), and mAvg 7-13 (OR=1.048, p=0.022). Children had modest PM_2.5_ association with first diagnosis during inversion season at mAvg 14-20 (OR=1.020, p=0.032) and during wildfire season at mAvg 14-20 (OR=1.059, p=0.040), with strong wildfire risk at mAvg 21-27 (OR=1.100, p=0.001). No association of PM_2.5_ with first COPD diagnosis was found in wildfire season, inversion season, or overall (Supplemental Figure S5B). Increases in ozone were not associated with any elevation in risk of new diagnosis of either asthma or COPD.

**Supplemental Discussion**

Age-stratified analyses revealed risk of asthma hospitalization in both adults and children, but with differential lag patterns. In wildfire season, risk in adults was elevated during the first 3 days after increases in PM_2.5_. Children, however, were not admitted until after a week or more, which may be due in part to physiological reasons and to behavioral factors that impede timely receipt of necessary care for children. Such age-based differences were previously reported for acute lower respiratory infections.(10) Sex-specific differences were also noted, with males experiencing greater and more prolonged risk of both asthma and COPD that were most notable during wildfire season. Biological mechanisms may in part account for this, but less cautious choices during wildfire smoke exposure may also have contributed. Importantly, that the associations of PM_2.5_ with outcomes in children were delayed compared to adults may reveal a potential greater need in more vulnerable populations for improved anticipatory care and for a better awareness of the risks during wildfire events to help modify behavior when physiological symptoms arise.

The potential interaction with higher risk for asthma admission among males with mAvg 21-27 exposure in wildfire season compared to females suggests that males may be more likely than females to delay the pursuit of healthcare for asthma in the wildfire season until four weeks after an acute elevation in PM_2.5_. In contrast, the noted differences in risk of COPD hospitalization at lag 0 for males compared to females suggests that males with COPD are more anxious to seek care immediately in the wildfire season when they experiences acute symptoms and females either delay seeking care or, so no significant elevation in COPD admission was seen among females, that females are more adept at resolving their COPD symptoms without assistance of medical professionals. Both of these findings for males suggest that additional support for males may be needed, either more encouragement for males regarding seeking care when asthma symptoms present or perhaps additional education regarding how to resolve both asthma and COPD symptoms using self-care techniques during wildfire events.

Short-term increases in ozone were not strongly associated with any outcome. Importantly, this study did not examine ozone associations with health outcomes in the warm season versus the cold season. These findings for ozone in the wildfire season from June through October may not compare directly to the analyses or results of other studies of ozone and health.

**Supplemental Reference**

1. Rosner B. Fundamentals of Biostatistics. 5th Ed. Pacific Grove, CA: Duxbury, 2000:243–244.

**Supplemental Table S1**. Odds ratios (with 95% confidence intervals) for the association of ambient ozone air pollution with hospitalization in an emergency or inpatient setting for the primary diagnosis of asthma or COPD.

**Wildfire Season Inversion Season All Months**

**Pollutant/Event Lag (June-October) (November-March) (January-December)**

*Ozone air pollution (ORs and 95% CI are per +10 ppb)*

Asthma Hospitalization (emergency or inpatient)

Lag 0 1.017 (0.981, 1.053) 1.010 (0.982, 1.039) 1.005 (0.985, 1.025)

Lag 1 1.019 (0.983, 1.056) 0.995 (0.967, 1.024) 0.992 (0.973, 1.012)

Lag 2 1.009 (0.973, 1.046) 0.979 (0.952, 1.007) **0.978** (0.959, 0.998)

mAvg 0-2 1.027 (0.980, 1.076) 1.003 (0.968, 1.040) 0.988 (0.964, 1.014)

mAvg 0-6 1.046 (0.981, 1.116) 0.958 (0.911, 1.006) **0.963** (0.932, 0.996)

mAvg 7-13 1.045 (0.979, 1.115) 0.975 (0.926, 1.025) 0.972 (0.940, 1.006)

mAvg 14-20 1.029 (0.967, 1.094) 0.982 (0.934, 1.032) 0.997 (0.964, 1.006)

mAvg 21-27 1.014 (0.953, 1.079) 1.014 (0.964, 1.067) 1.009 (0.975, 1.043)

COPD Hospitalization (emergency or inpatient)

Lag 0 0.999 (0.940, 1.063) 0.997 (0.950, 1.045) 0.998 (0.965, 1.033)

Lag 1 0.957 (0.900, 1.017) 0.966 (0.921, 1.013) 0.973 (0.941, 1.006)

Lag 2 0.971 (0.913, 1.032) 0.997 (0.951, 1.045) 0.985 (0.952, 1.018)

mAvg 0-2 0.952 (0.878, 1.032) 0.985 (0.927, 1.047) 0.976 (0.935, 1.020)

mAvg 0-6 0.950 (0.849, 1.062) 0.978 (0.900, 1.063) 0.962 (0.909, 1.019)

mAvg 7-13 0.989 (0.882, 1.110) **1.099** (1.010, 1.195) **1.067** (1.007, 1.131)

mAvg 14-20 1.008 (0.903, 1.125) 0.982 (0.903, 1.069) 0.987 (0.932, 1.045)

mAvg 21-27 1.048 (0.940, 1.168) 0.943 (0.865, 1.028) 0.980 (0.925, 1.038)

All odds ratio and 95% confidence interval data are per +10 ppb for ozone.

Results that are bolded had p≤0.05 and p>0.025 and were not considered significant after correction for multiple comparisons.

**Supplemental Figure S1**. Air pollution data during the study period (January 1999-March 2022): a) for ozone demonstrated a trend toward higher levels in the last 10 years (note that ozone was only measured in warm months until 2006 and, thus, ozone analyses for inversion season and for all months of the year only included events from 2006-2022; green line: threshold for elevated ozone used in panel b). Gray data points are measured pollution levels from April and May. Evaluation of thresholds of air pollution showed the trends over the years to have more days with: b) short-term increases of ozone during wildfire season (with minimal changes in elevated ozone during inversion season).

a)


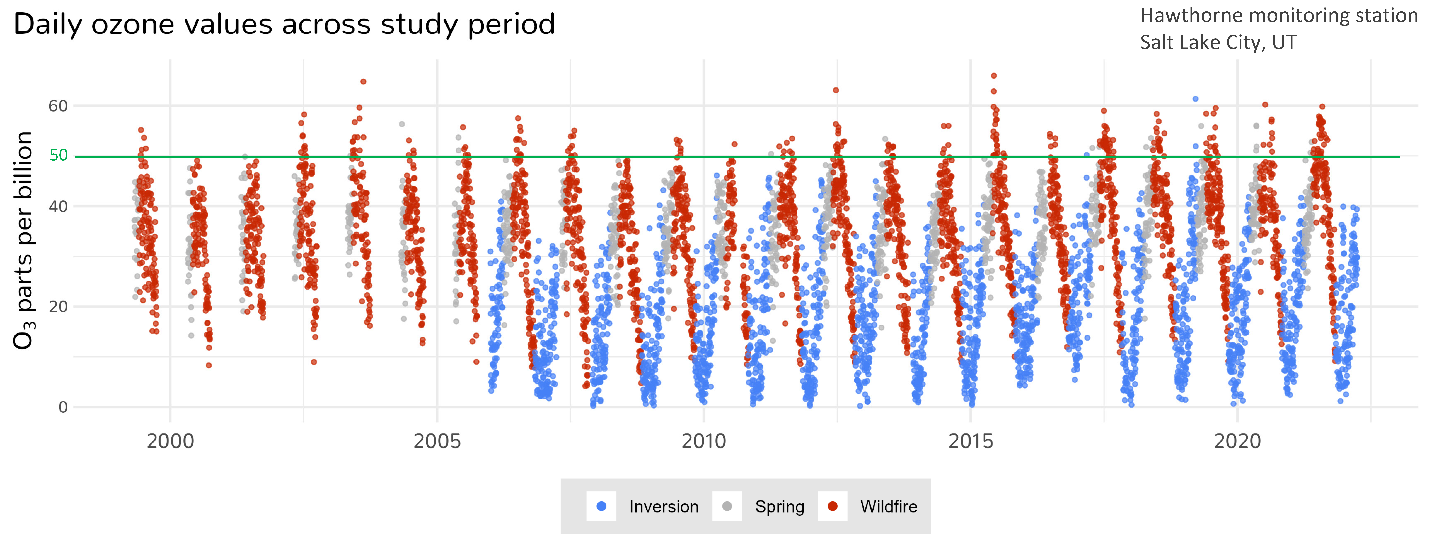


b)


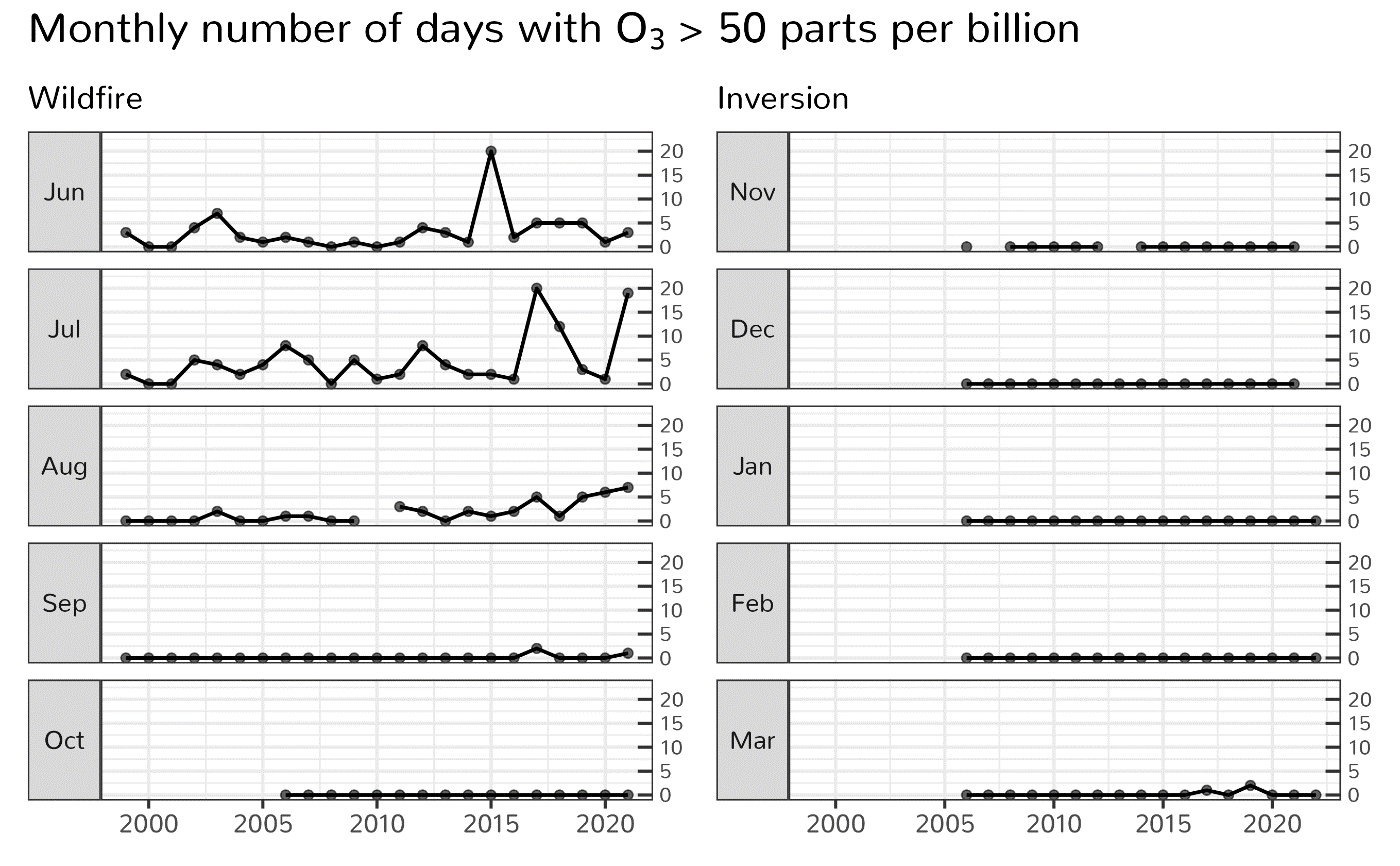


**Supplemental Figure S2**. Forest plots for a) adults aged ≥18 years and b) children aged <18 years that display the association of short-term increases in PM_2.5_ with hospitalization for asthma during wildfire season (red) and inversion season (blue). Data are OR (diamonds) and 95% CI (whiskers). The x-axis provides the various days of lag examined between exposure and events.

1. Adults


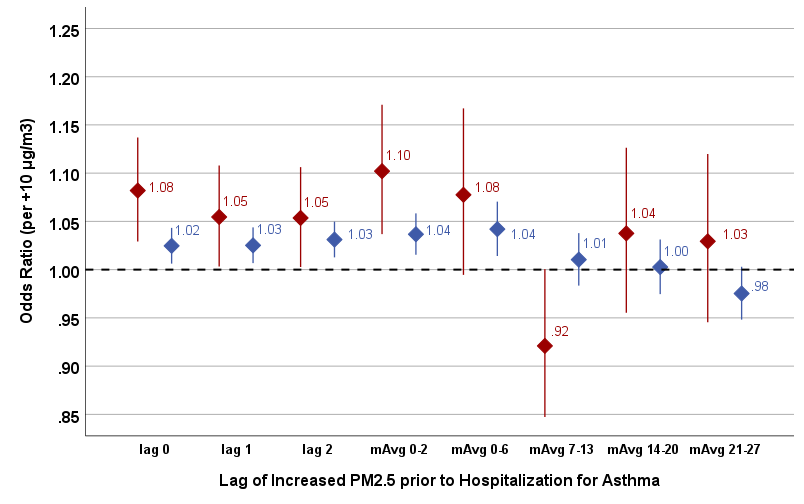


1. Children


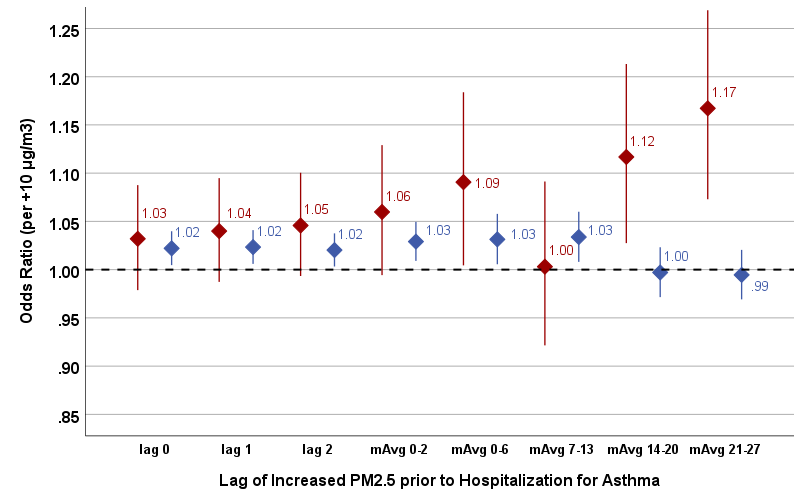


**Supplemental Figure S3**. Forest plots for the association of short-term increases in PM_2.5_ and asthma hospitalization for: a) females and b) males during wildfire season (red) and inversion season (blue), and the association of PM2.5 and COPD hospitalization for: c) females and d) males. Data are OR (diamonds) and 95% CI (whiskers). The x-axis provides the days of lag examined between exposure and events.

1. Females (asthma hospitalization)


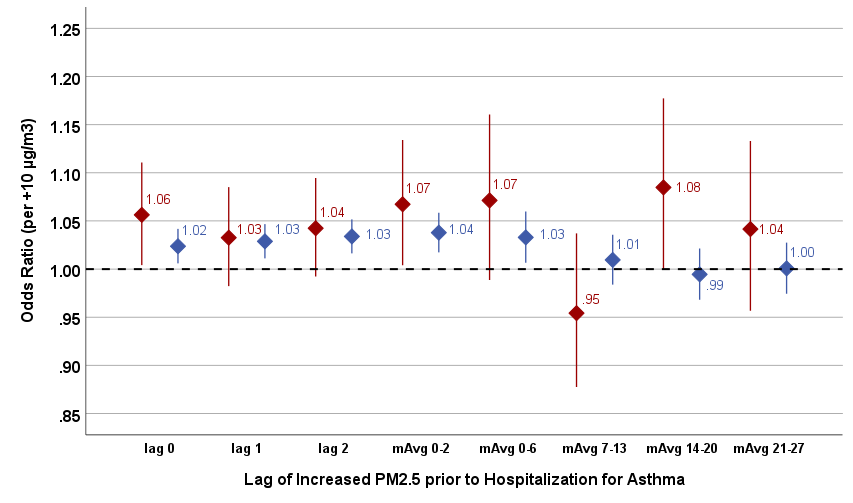


1. Males (asthma hospitalization)


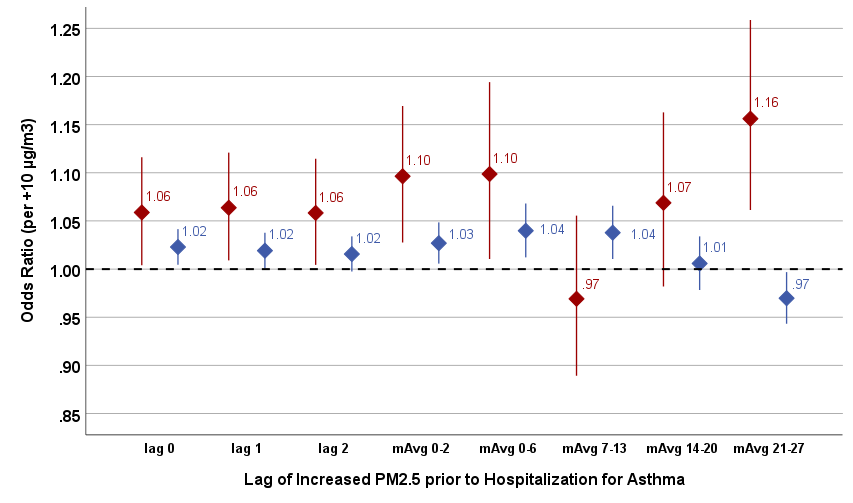


1. Females (COPD hospitalization)


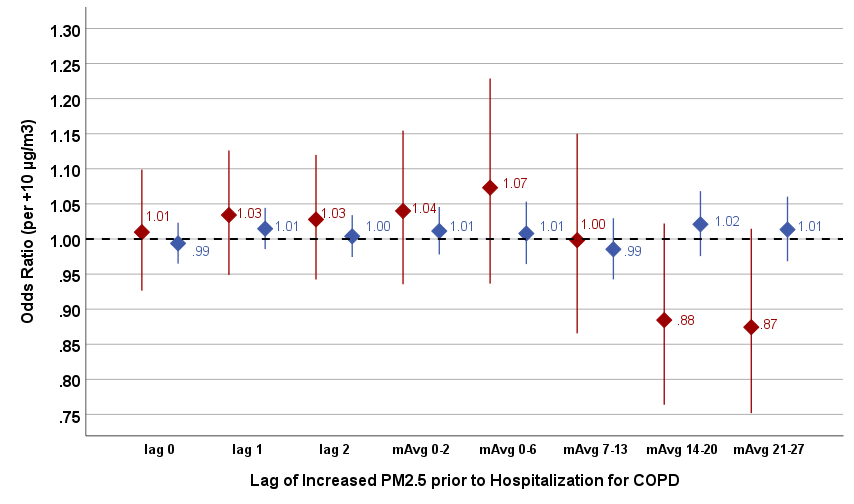


1. Males (COPD hospitalization)


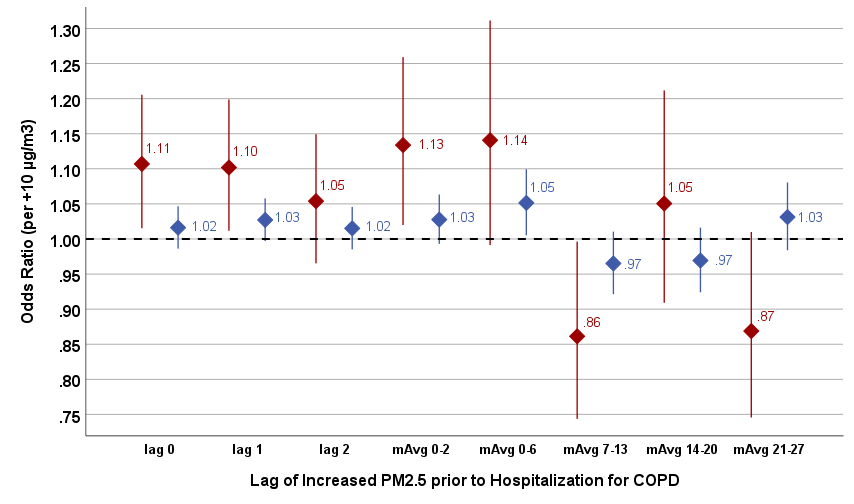


**Supplemental Figure S4**. Association of short-term increases in ozone with hospitalization for: a) asthma or b) COPD, in wildfire (red) or inversion (blue) seasons. Data are OR (diamonds) and 95% CI (whiskers).

a)


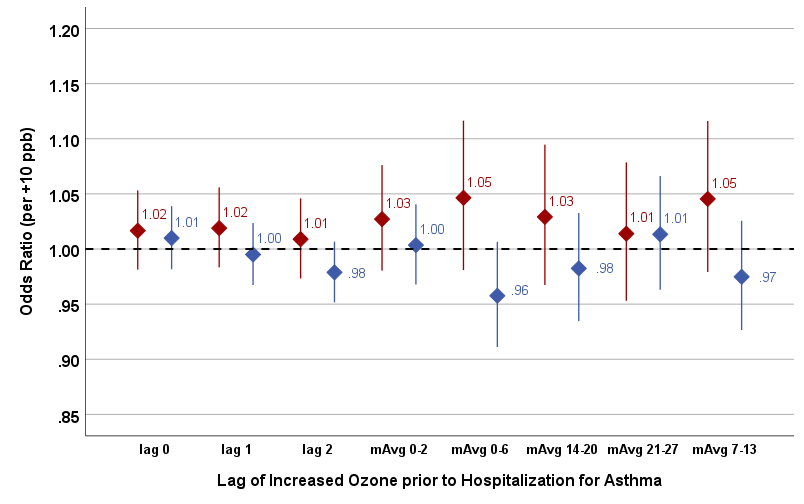


b)


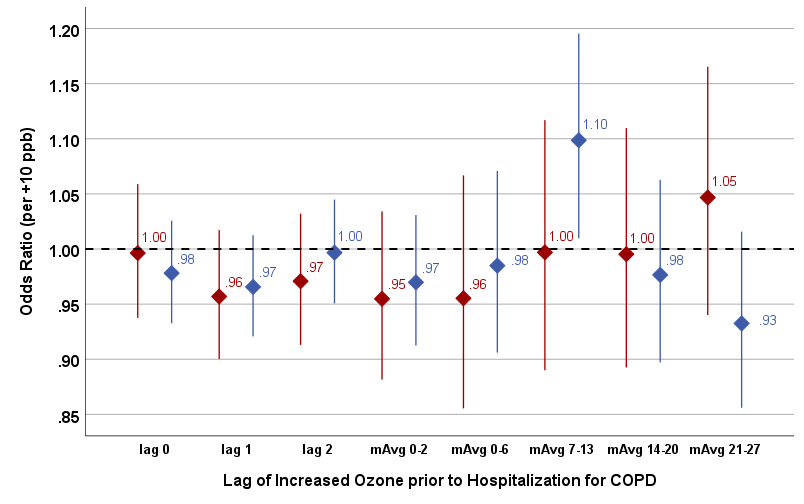


**Supplemental Figure S5**. Association of short-term increases in PM_2.5_ with first diagnosis of: a) asthma, or b) COPD, in wildfire (red) or inversion (blue) seasons. Data are OR (diamonds) and 95% CI (whiskers).

a)


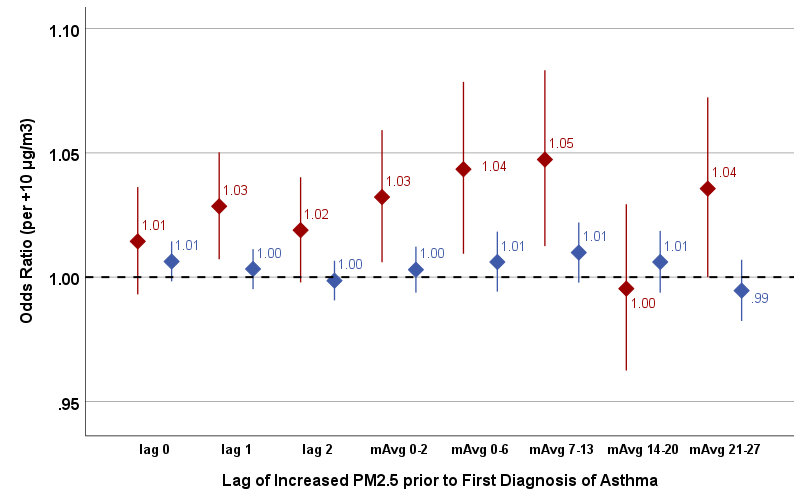


b)


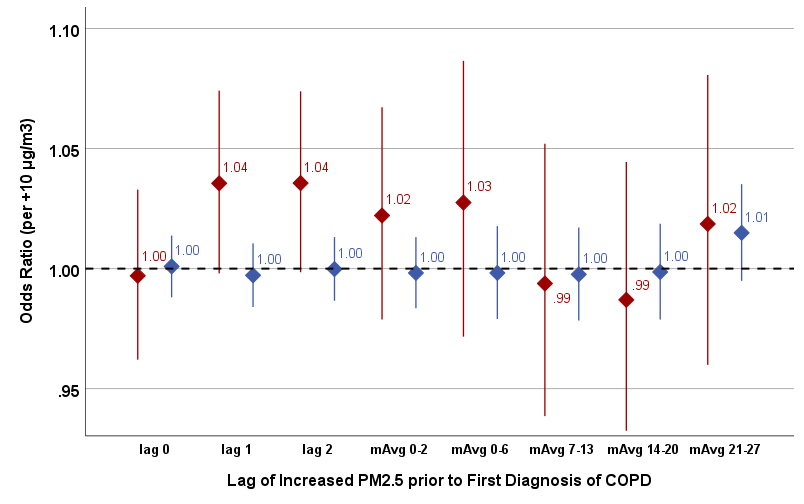

Supplement: Supplementary Data [file mmc1.docx]
